# Focusing on Selinexor for Holding and Bridging Prior to CAR-T in Relapsed/Refractory Multiple Myeloma

**DOI:** 10.3390/jcm14124071

**Published:** 2025-06-09

**Authors:** Jack Khouri, Douglas Sborov, Adriana Rossi, Thomas Martin, Trinayan Kashyap, Tomer Mark, Muhamed Baljevic

**Affiliations:** 1 Department of Hematology and Medical Oncology, Cleveland Clinic, Cleveland, OH 44195, USA; khourij@ccf.org; 2 University of Utah Huntsman Cancer Institute, Salt Lake City, UT 84112, USA; douglas.sborov@hci.utah.edu; 3 Icahn School of Medicine at Mount Sinai, New York, NY 10029, USA; adriana.rossi@mssm.edu; 4 Helen Diller Family Comprehensive Cancer Center, San Francisco Medical Center, University of California, San Francisco, CA 94143, USA; tom.martin@ucsf.edu; 5 Karyopharm Therapeutics, Newton, MA 02459, USA; tkashyap@karyopharm.com (T.K.); tomer.mark@karyopharm.com (T.M.); 6 Vanderbilt University Medical Center, Nashville, TN 37232, USA

**Keywords:** selinexor, CAR-T, BCMA, bridging, T-cell exhaustion

## Abstract

**Background:** The remarkable efficacy of B-cell maturation antigen (BCMA)-directed chimeric antigen receptor T-cell therapy (CAR-T) has had a significant impact on treatment strategies for relapsed/refractory multiple myeloma (RRMM). However, response durability remains a concern, necessitating the optimization of CAR-T procedures. Therapies preceding CAR-T therapy are crucial for disease control and preserving T-cell fitness. **Methods:** This review summarizes the evidence supporting the potential of selinexor-based regimens as holding or bridging therapy with preclinical research, demonstrating selinexor’s ability to foster an anti-inflammatory tumor microenvironment. **Results:** Selinexor enhances CD8+ T-lymphocyte and NK cell activation, re-polarizes macrophages, and inhibits immunosuppressive cells. Bone marrow samples from patients in clinical studies show that selinexor increases CD8 and granzyme B expression in T-cells. Selinexor also disrupts NK cell inhibition, enhances anti-tumor activity, and reduces pro-inflammatory cytokines. Selinexor may upregulate BCMA expression and increase myeloma cell immunogenicity. Real-world data suggests selinexor as bridging therapy does not compromise CAR-T outcomes and may even improve them. **Conclusions:** Overall, the evidence indicates selinexor’s potential to optimize CAR-T outcomes, warranting further investigation as a holding or bridging therapy for CAR-T.

## 1. Introduction

The introduction of B-cell maturation antigen (BCMA)-directed chimeric antigen receptor T cell therapy (CAR-T) in clinical practice has radically shifted the treatment paradigm of relapsed/refractory multiple myeloma (RRMM). At present, two commercial products have been approved for routine use in RRMM patients with early and late relapse: idecabtagene vicleucel (ide-cel) and ciltacabtagene autoleucel (cilta-cel). While autologous CAR-T therapy has demonstrated remarkable efficacy in clinical trials and real-world analyses for RRMM, treatment is not curative and response durability is a concern. Additionally, early non-relapse mortality events observed after CAR-T further highlight the need to optimize the phases and procedures surrounding CAR-T use, including holding therapy, leukapheresis, bridging, lymphodepletion, and cell reinfusion. Various factors have been identified that may compromise clinical outcomes following T cell redirecting therapies, such as age, performance status, cytogenetic abnormalities, the presence of extramedullary disease, prior treatments, and immunologic function, among others [1,2,3,4].

Holding therapy (prior to apheresis) or bridging therapy (post apheresis, but prior to CAR-T infusion) is a plasma cell-directed therapy preceding CAR-T treatment which plays a critical role in the overall effectiveness of CAR-T use by providing disease control for a long enough duration to allow successful CAR-T manufacturing. Of utmost importance is preserving endogenous T-cell fitness without compromising cytotoxic T-cell function in the tumor immune microenvironment. Optimizing bridging therapy remains elusive due to a lack of prospective evidence to support specific recommendations; however, expert panels from the American Society for Transplantation and Cellular Therapy in 2024 and the International Myeloma Working Group in 2025 have issued opinions suggesting that selinexor-based regimens should be considered as holding therapy (prior to apheresis) or bridging to CAR-T [5,6].

**Figure 1:**
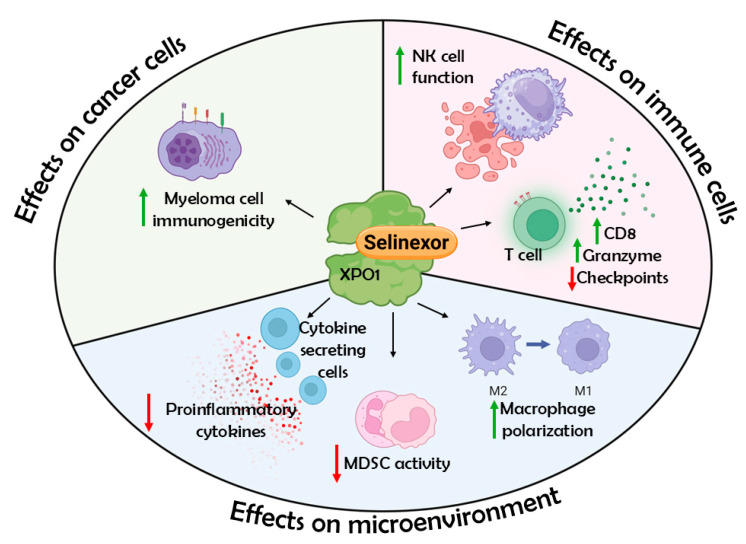
Overview of selinexor-mediated immunomodulation. Selinexor-mediated inhibition of XPO1 activity is associated with increased antibody-directed cytotoxicity of NK cells against cancer target cells and associated with increased CD8 and granzyme B expression of CD3+ T-cells without induction of immune checkpoints. XPO1 inhibition can reduce proinflammatory cytokine signaling (often through the NF-κB pathway), lower MDSC immunosuppressive activity by transforming MDSCs into neutrophil-like cells, and polarize macrophages towards M1 and away from a tumor-promoting M2 state. In addition to inducing cell cycle arrest and apoptosis of cancer cells, selinexor is also associated with enhanced immunogenicity of surviving myeloma cells. Created with Biorender.com.

Pre-clinical studies have shown that selinexor, an exportin 1 (XPO1) inhibitor, acts as an effective anti-cancer agent and fosters an anti-inflammatory tumor immune microenvironment without harming endogenous tumor-surveillance mechanisms [7,8]. Blocking XPO1 leads to the activation of CD8+ T-lymphocytes and NK cells, M2 to M1 macrophage re-polarization, and the inhibition of myeloid-derived suppressor cells (MDSCs) and neutrophil extracellular traps, which provide potent immunosuppressive effects in the tumor microenvironment (Figure 1). In a series of early and later phase studies, selinexor in various regimens demonstrated feasibility and efficacy in RRMM, including in patients with heavily pretreated disease [9,10,11,12,13,14,15].

## 2. Methods

The direct impact of selinexor on T cells was evaluated in patients from the STOMP study (NCT02343042) treated with selinexor triplet therapies [16]. The median time on treatment for the cohort of patients included in the analysis was 230 days. When compared to pre-selinexor exposure, the CD3+ cells in the bone marrow samples from patients who underwent selinexor-based treatment showed an increased expression of CD8 and granzyme B, consistent with the activation of cytotoxic T cells. Importantly, exhaustion markers such as LAG3, PD-1, and CTLA4 were not expressed on T cells post selinexor-triplet therapy, even in samples from patients who had lost clinical response, indicating that the mechanism of resistance to selinexor does not involve T-cell exhaustion [16]. Supporting this observation in the STOMP patient cohort, a preclinical study found that clinically relevant weekly or twice weekly dosing of selinexor maintained normal CD8+ T-cell functioning, and there was an observed decrease in markers of T-cell exhaustion in treated mice. Specifically, in a B16 melanoma mouse model, decreases in LAG3 and PD-1 were observed on tumor infiltrating CD8 T cells in mice treated with biweekly selinexor compared to vehicle [8].

NK cell dysfunction has been reported in multiple human cancers and has been linked to more aggressive MM [17]. Like T cells, NK cell function can be suppressed by immune checkpoint expression on target cells. The Expression of Human Leukocyte Antigen E (HLA-E) on target cells leads to NK cell inhibition through interaction with the inhibitory receptor NKG2A on NK cells, enabling tumor cell escape from immune surveillance. Selinexor has been shown to disrupt the inhibitory NKG2A:HLA-E axis to activate NK cells against cancer. XPO1 inhibition by selinexor selectively reduced the surface expression of HLA-E on cancer cells, leading to functional increases in the antibody-dependent cellular cytotoxicity of the NKG2A+ cells against the tumor [18]. In addition to NK cells, the inhibition of NKG2A has also been shown to enhance the efficacy of cancer vaccines in murine tumor models via CD8+ T cell activation [19].

## 3. Results

Selinexor also impacts the differentiation and suppressive functions of MDSCs, providing a potential mechanism for limiting the immunosuppressive effects of the tumor microenvironment [20]. Selinexor treatment of mouse models of leukemia and lymphoma reduced the ability of MDSCs to suppress proliferation of CD4+ and CD8+ T cells. The XPO1 blockade promoted the differentiation of MDSCs into T-cell-activating neutrophil-like cells, which enhanced the overall antitumor immune response and restrained tumor growth. While the mechanism of action remains to be fully elucidated, XPO1 inhibition leads to the nuclear entrapment of ERK1/2, resulting in the prevention of ERK1/2 phosphorylation following the IL-6-mediated activation of the MAPK signaling pathway. The disruption of this pathway suppresses the pro-inflammatory tumor microenvironment, limiting T-cell exhaustion [20]. The group of Jimenez et al. explored the effect of selinexor and the BTK inhibitor ibrutinib on the polarization of macrophages in a CNS lymphoma model [21]. The study revealed that selinexor favored an anti-tumor innate immune response by shifting polarization from the M2 subtype that is associated with wound repair to the inflammatory M1 subtype. Selinexor was shown to have a stronger effect than ibrutinib on the M2 -> M1 re-polarization, with the combination showing no difference compared to selinexor alone. Selinexor has also been shown to reduce the plasma levels of many key pro-inflammatory cytokines (e.g., IL-6, TGFβ, IL-1β, IL-10, IFN-γ, TNF-α) in patients with myelofibrosis [22], and also in ex vivo studies with PBMCs [23]. The reduction in the levels of most of the cytokines is driven by the selinexor suppression of NF-κB transcriptional activity [24]. Additionally, in vitro studies have demonstrated selinexor’s inhibitory effect on the formation of neutrophil extracellular traps (NETs), net-like structures composed of externalized DNA–histone complexes and proteins released by neutrophils [25]. Since neutrophil recruitment to the tumor microenvironment and release of extracellular traps have been shown to promote tumor growth and metastasis, the inhibition of NETs may contribute to selinexor’s overall anticancer mechanism of action [26].

Low-dose selinexor was also reported to be associated with the upregulation of BCMA expression on plasma cell lines and subsequently linked to the enhanced function of CAR-T cells in vitro, further supporting the potential use of selinexor for bridging therapy prior to anti-BCMA-directed CAR-T [27]. Furthermore, there is initial evidence suggesting that prolonged exposure to selinexor can lead to the increased immunogenicity of MM cells mediated by the expression of the Enhancer of Zeste homolog 2 (EZH2) and its catalytic role on the tri-methylation of histone H3 at Lys 27 (H3K27me3) to regulate gene expression. EZH2 expression is found to positively correlate with selinexor’s ex vivo sensitivity in MM patient samples [28]. Likewise, transcriptional profiling with paired ex vivo sensitivity assays of primary MM samples showed that selinexor silences the H3K27me3-regulated genes involved in antigen presentation and that acquired selinexor resistance correlated with the expression of immunogenic targets [28].

There is growing clinical evidence supporting the use of selinexor prior to CAR-T infusion. Real-world data reported by the US Myeloma Immunotherapy Consortium found that patients with RRMM who received selinexor as bridging therapy prior to ide-cel experienced a progression-free survival (PFS) of 9.8 months (95% CI: 4.4–13.9) that was not statistically different than an immunomodulatory drug (IMiD)-based bridge. The use of bridging therapy in the entire study cohort demonstrated a median overall survival (OS) of 13.8 months (95% CI, 11.97-NR) for the group that received bridging therapy vs. not reached in the no bridging therapy group (*p* = 0.002), likely due to a combination of patient-, disease-, and therapy-related factors [4]. These higher-risk patients have the greatest need for additional strategies to mitigate any added adverse impact of bridging therapy choice on CAR-T clinical outcomes. An additional real-world evidence study of heavily pretreated patients with RRMM who received a selinexor-containing regimen (in the median seventh line of therapy) prior to BCMA-directed CAR-T (in the median ninth line of therapy) experienced a median PFS and OS post CAR-T administration of 8.0 (IQR: 3.1 to 39.5) and 35.9 months (IQR: 14.2 to not reached), respectively [29]. The most frequently used selinexor regimens were bortezomib/dexamethasone (28.9%) and carfilzomib/dexamethasone (20%). Regimens with anti-CD38 monoclonal antibodies or immunomodulatory drugs were rarely used. The majority of patients (75.6%) started at a selinexor dose of 80 mg or less weekly. Patients who received a selinexor-based regimen immediately prior to CAR-T had a reduced risk of disease progression (HR = 0.40; 95% CI: 0.14 to 1.09) and death (HR = 0.08; 95% CI: 0.02 to 0.46). A retrospective study of 7 patients who received CAR-T and were treated with a selinexor-based regimen immediately prior to apheresis demonstrated an overall response rate of 100%, with all 7 patients achieving very good partial response or better [30]. The selinexor regimens included carfilzomib and dexamethasone (4 patients), daratumumab and dexamethasone (2 patients), and pomalidomide and dexamethasone (1 patient). A retrospective analysis of RRMM patients who were treated with ide-cel showed that bortezomib as a bridging therapy led to a significantly shorter PFS compared to no bridging. Additionally, bortezomib also led to a decrease in the CD4:CD8 ratio and increase in CD4+ and CD8+ T-cell inhibitory markers in CD138-negative cells from bone marrow mononuclear cells, collected post-apheresis, but pre-lymphodepletion, which has also been associated with a shorter PFS to ide-cel. Intriguingly, the selinexor-associated modulation of the T cell repertoire was able to rescue the negative effects of bortezomib on ide-cel outcomes in the selinexor/bortezomib group [28]. Accounting for the inherent limitations of small sample sizes and retrospective designs, the studies did control for known prognostic variables in their statistical analyses. To sum, selinexor at a minimum does not appear to compromise T-cell-based therapy outcomes, and there is value in further exploring whether selinexor can enhance T-cell fitness and result in manufacturing more effective CAR-T cells. Prospective clinical trials aimed at assessing selinexor’s potential in this context are warranted.

## 4. Conclusions

The multiple reports of effects promoting T and NK cell function as well as the inhibition of MDSC function and levels of pro-inflammatory cytokines supported by real-world evidence of selinexor’s utility prior to CAR-T suggest that selinexor could improve the tumor microenvironment and, therefore, optimize CAR-T anti-myeloma efficacy. Based on pre-clinical evidence of T-cell impact by XPO1 inhibitors, a rational consideration of selinexor combination regimens could support T-cell fitness and minimize T-cell exhaustion [31], paving the path toward an optimized priming of T-cell repertoire, more durable CAR-T activity, and better long-term patient outcomes. As such, selinexor-based regimens should be further explored for patients with RRMM destined to receive CAR-T, in particular, as holding therapy and, if needed, as a part of bridging therapy. There also could be potential beyond MM, but that has not been explored.

## Data Availability

No new data were created.

## Abbreviations

BCMA	B-cell maturation antigen
CAR-T	Chimeric antigen receptor T cell therapy
cilta-cel	Ciltacabtagene autoleucel
CNS	Central nervous system
EZH2	Enhancer of Zeste homolog 2
H3K27me3	Histone H3 at Lys 27 (tri-methylation)
HLA-E	Human Leukocyte Antigen E
ide-cel	Idecabtagene vicleucel
IMiD	Immunomodulatory drug
IQR	Interquartile range
MDSCs	Myeloid-derived suppressor cells
MM	Multiple myeloma
NETs	Neutrophil extracellular traps
ORR	Overall response rate
OS	Overall survival
PBMCs	Peripheral blood mononuclear cells
PFS	Progression-free survival
RRMM	Relapsed/refractory multiple myeloma
XPO1	Exportin 1
